# Toll-like receptor 9 (TLR9) polymorphism associated with symptomatic malaria: a cohort study

**DOI:** 10.1186/1475-2875-11-168

**Published:** 2012-05-17

**Authors:** Ahmeddin H Omar, Michio Yasunami, Akiko Yamazaki, Hiroki Shibata, Michael F Ofori, Bartholomew D Akanmori, Mohammed Nasir Shuaibu, Mihoko Kikuchi, Kenji Hirayama

**Affiliations:** 1 Department of Immunogenetics, Institute of Tropical Medicine (NEKKEN) and Global COE Program, Nagasaki University, 1-12-4 Sakamoto, Nagasaki, 852-8523, Japan; 2 Noguchi Memorial Institute for Medical Research, University of Ghana, Legon, Ghana; 3 Center for International Collaborative Research (CICORN), Nagasaki University, 1-12-4 Sakamoto, Nagasaki, 852-8523, Japan

**Keywords:** Cohort study, TLR9, Symptomatic malaria, Genetic susceptibility, Genetic polymorphism, Haplotype, Luciferase promoter assay

## Abstract

**Background:**

In areas mesoendemic for malaria transmission, symptomatic individuals play a significant role as reservoirs for malaria infection. Understanding the pathogenesis of symptomatic malaria is important in devising tools for augmenting malaria control. In this study, the effect of TLR9 polymorphisms on susceptibility to symptomatic malaria was investigated among Ghanaian children.

**Methods:**

Four hundred and twenty nine (429) healthy Ghanaian children, aged three to eleven years (3–11 years), were enrolled into a cohort study and actively followed up for symptomatic malaria for one year. Four TLR9 single nucleotide polymorphisms (SNPs) namely: rs187084 (C-1486 T), rs5743836(C-1237 T), rs352139 (G + 1174A) and rs352140 (G + 2848A) were genotyped by direct sequencing, and their attributable and relative risks for symptomatic malaria determined. TLR9 haplotypes were inferred using the PHASE software and analysed for the risk of symptomatic malaria. A luciferase assay was performed to investigate whether the TLR9 haplotypes influence TLR9 promoter activity.

**Results:**

The rs352139 GG genotype showed a significantly increased relative risk of 4.8 for symptomatic malaria (*P* = 0.0024) and a higher mean parasitaemia (*P* = 0.04). Conversely, the rs352140 GG genotype showed a significantly reduced relative risk of 0.34 (*P* = 0.048). TLR9 haplotypes analyses showed that TTAG haplotype was significantly associated with reduced relative risk of 0.2 for symptomatic malaria (*P* = 4×10^-6^) and a lower mean parasitaemia (0.007), while CTGA haplotype had an increased relative risk of 3.3 (*P* = 0.005). Functional luciferase reporter gene expression assay revealed that the TTA haplotype had a significantly higher promoter activity than the CCG, CTG and TCG haplotypes.

**Conclusions:**

Taken together, these findings indicate a significant association of TLR9 gene polymorphisms with symptomatic malaria among Ghanaian children in Dangme-West district.

## Background

Despite the tremendous achievements in malaria control over the past few years, malaria still remains a major public health problem in the endemic countries. According to the WHO world malaria report, there were an estimated malaria cases and deaths of 225 million and 781,000, respectively, in the year 2009 [1]. Malaria has a wide spectrum of infections that ranges from asymptomatic malaria, symptomatic (mild) malaria, to severe (complicated) malaria infections. Although severe malaria, which constitutes 1-2% of all malaria cases, is responsible for most of the malaria deaths, symptomatic malaria accounts for the major burden of the disease’s morbidity [2]. Moreover, while asymptomatic gametocyte carriers are the major transmission reservoir in areas hyperendemic for malaria [3], symptomatic individuals play an increased role as reservoir for malaria infections in areas with low intensity of malaria transmission [4].

Among the factors that have been postulated to determine the clinical outcome of malaria infections are host genetic make-up and immunity [5]. The contribution of genetics in susceptibility to malaria has been well documented, with the sickle cell trait, haemoglobin C variants and glucose-6-phosphate dehydrogenase deficiency conferring protection against severe malaria [6]. On the other hand, α-thalassaemia shows a more complex effect of increasing susceptibility to mild malaria while conferring protection against severe malaria [7]. Thus, it is possible that the mechanism responsible for protection against mild and severe malaria may be different from each other. This hypothesis has been reinforced by the fact that in malaria endemic areas, immunity to severe malaria is achieved at a time when susceptibility to mild malaria is still high [8].

Toll like receptors (TLR) are innate immune receptors that recognize pathogen associated molecular patterns and trigger activation of the intracellular signal cascade that induces transcription of inflammatory cytokines, type 1 interferons and chemokines [9]. In addition, stimulation of TLRs also leads to dendritic cell maturation and induction of adaptive immune response [10]. TLR2 and TLR4 have been reported to recognize *Plasmodium falciparum* glycosylphosphatidylinositol (GPI) [11], while TLR9 has been reported to recognize malaria haemozoin [12] and/or *Plasmodium* DNA-bound haemozoin [13].

TLR9-encoding gene is located on chromosome 3p21.3 and spans approximately 5 kb. It consists of 2 exons and encodes 1032 amino acids [14]. The TLR9 gene has been implicated in pathogenesis of severe malaria both in murine model and in humans. In animal studies, it has been demonstrated that mice deficient in TLR9 survived cerebral malaria better than the wild type mice [15] and inhibition of TLR9 activations by TLR9 agonist conferred protection against cerebral malaria in mice [16]. A number of single nucleotide polymorphisms (SNPs) for TLR9 have been identified, and four of these SNPs; rs187084, rs5743836, rs352139 and rs352140, have been reported to show high heterozygocity among three major US ethnic groups [17]. Several studies have focused on the relationship of TLR9 polymorphisms and severe malaria phenotypes, with some reporting positive association. For example, in a study in Ghana on malaria in pregnancy (n = 304), the TLR9 promoter polymorphism rs187084 C allele was associated with increased risk of low birth weight among term infants [18], while a Brazilian study among adults (n = 304) with mild malaria associated the promoter polymorphisms rs187084 C allele and rs5743836 C allele with high parasitaemia (>10,000 parasite/μl) [19]. However, these effects of TLR9 promoter polymorphisms on severe malaria phenotypes were not replicated in a large family and population-based association study from Malawi and Gambia (n > 6000) that found no convincing association of the four common TLR9 SNPs with severe malaria [20]. On the other hand, few studies have focussed on the effect of TLR9 polymorphisms on susceptibility to mild malaria. Two studies, carried out in Brazil [19] and Iran [21], reported no influence of TLR9 promoter polymorphism on susceptibility to mild malaria in their respective populations. However, the effect of TLR9 polymorphisms on mild malaria among the African population is not well understood.

In the present study, healthy Ghanaian children living in an area mesoendemic for malaria transmission were recruited into a prospective cohort study for one year, and the effect of the TLR9 SNPs on susceptibility to symptomatic malaria investigated.

## Methods

### Study area and subjects

Details about the study area, population, set-up of the cohort and the follow-up protocol has previously been described [22]. In brief, the cohort study was conducted at Asutuare, a sub-district of Dangme-West District in the Great Accra region of Ghana. The district has two rainy seasons in a year, April to July and October to December, and consequently malaria transmission is seasonal, with the peak transmission coinciding with the period of the major rainy seasons while the dry seasons having low malaria transmission [23]. It is estimated that individuals in Dodowa, (the district headquarter for Dangme-West district), are exposed to about 20 infective bites per year, and 98% of the infections are due to *P. falciparum*[23]. Four hundred and twenty nine (429) healthy Ghanaian children, aged 3–11 years, were enrolled into a one-year prospective cohort study, from June 2007 to July 2008, which spanned across three (3) rainy seasons. Only one child per household was recruited in order to avoid inclusion of closely related individuals. The study participants were actively followed up for clinical malaria symptoms with regular home visits of two-week intervals. During the visits, data on the health status of the participants for the previous two weeks were collected using a standard questionnaire and their body temperatures measured. Individuals suspected of clinical malaria were referred to the community health centre where medical examinations and blood smear for malaria were carried out and treatment given according to the recommendation of the Ghanaian Ministry of Health.

### Phenotype definition

In this study, a participant was considered to be suffering from symptomatic mild malaria, if he or she had a temperature that is greater than or equal to 38.0°C, with a parasite load that is greater than or equal to 5,000 parasite per microliter (μl) of blood. Informed consent was obtained from each participant’s parents/guardian after a detailed explanation of all procedures in the study. The study was approved by the Ghanaian Ministry of Health, Institutional Review Board (IRB) of Noguchi Memorial Institute of Medical Research, University of Ghana and IRB of Institute of Tropical Medicine, Nagasaki University.

### DNA extraction and polymerase chain reaction (PCR)

Sample collection and storage has been described in detail elsewhere [22]. Genomic DNA was extracted from blood sample using QIAamp DNA blood mini kit (Qiagen, Tokyo, Japan).

The PCR amplification for the rs187084, rs5743836, and rs352139 were carried out using the forward primer 5′-CTGTGGACATCGATATCGGTGT-3′ (PF3) and reverse primer 5′-AAGCTTCGCTGCGGCAGAAACCCTGT-3′ (i1R), while for rs352140; forward primer 5′-TCTAGACATCATGCTGGCCATGACC-3′ (2 F8) and reverse primer 5′-CAGAGCCACTCAACAGTGGACT-3′ (2R5) were used. Each PCR reaction contained 5.0 μl of 2xKOD FX buffer, 2.0 μl of 2 mM dNTP, 0.4 μl of forward primer, 0.4 μl of reverse primer, 0.2 μl of KOD Polymerase, 1.5 μl of water, and 0.5 μl of DNA and the PCR conditions used were as follows: One cycle at 95°C for 5 min, 35 cycles at 95°C for 1 min, 35 cycles at 64°C for 1 min, and one cycle at 72°C for 4 min.

### SNP genotyping

The TLR9 SNPs studied were identified by analysis of direct nucleotide sequencing of PCR products. The sequencing was carried out using the 3730 DNA Analyzer Applied Biosystems sequencing machine with the sequencing primers 5′-GGGTGTACATAATTCAGCAG-3′, 5′-GGCAAAGGAGCTCAGGAGTG-3′, 5′-GGAAGAACTTCTGCAGGTAG-3′, and 5′-GGAGAAGGTCTGGCTGCAG-3′ for rs187084, rs5743836, rs352139 and rs352140, respectively. The sequencing data was analysed using sequence scanner version 1.0, Applied Biosystems.

### Plasmid construction for luciferase gene reporter assay

**Figure 1:**
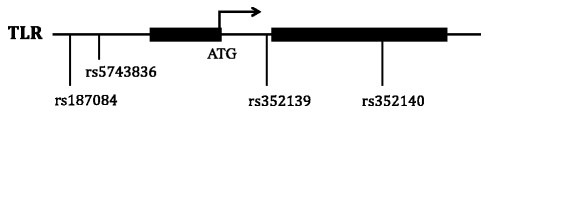
**Luciferase reporter activity of TLR9 (promoter-intron) plasmid constructs.a**), schematic of four reporter gene constructs that contains TLR9 promoter- intron region with TTA, CCG, CTG and TCG at position −1486, -1237, and +1174 polymorphic sites, respectively. **b**), luciferase expression of the four constructs in THP-1 cells and pGL4.10 as negative control. The luciferase activity levels are mean values of three (3) independent experiments that were all carried out in triplicate. Anova test and t-test were used to test for statistical significance. P value of <0.05 was considered to be statistically significant.

Using the PCR primers PF3 and i1R (named above) a DNA fragment, nucleotide positions −2268 to +1233 (NM_17442), which encompassed the promoter, exon 1 and intron 1 regions of the TLR9 gene was amplified from genomic DNA of four selected study subject. The four subjects were homozygous carriers of TTA, CTG, TCG, and CCG for SNPs at positions −1486, -1237 and +1174, respectively. Topo cloning of the PCR products were carried out using Zero blunt® Topo® cloning kit and transformed into One Shot® TOP10 Competent cells. Positive clones were selected, plasmid extracted and presence of the expected fragment confirmed by restriction digestion analysis and DNA sequencing. The cloned fragment was then treated with BamHI, HindIII and BclI restriction enzymes to obtain a 2754-bp DNA fragment, nucleotide position (−1521 to +1233). This fragment was then cloned into BglII and HindIII restriction sites of the firefly luciferase reporter vector pGL4.10 (Promega, Madison, WI) resulting in construction of four (4) promoter-intron plasmids (Figure 1a). The presence of the insertions and their orientation were confirmed by restriction digestion analysis and DNA sequencing.

### Transient transfection assay

THP-1 (human acute monocytic leukaemia) cells were obtained from the American type culture collection (ATCC®) and cultured as per their instructions in RPMI-1640 medium supplemented with 10% foetal calf serum (FCS) and Antibiotic-Antimycotic, 100X (AAS) (Gibco, Life technologies). After more than 90% confluence was achieved in a cell culture flask, 1 × 10^6^ cells/well were then seeded into a 24-well plates; and transiently transfected with 2 μg of each of the four TLR9 plasmid constructs (designated as TTA, CCG, CTG and TCG promoter-intron plasmids) and 2 μg of an empty pGL4.10 vector (promega) as a negative control. All the transfections were performed using Fugene HD® (Promega) according to the manufacturer’s recommendations. After 48 h of incubation, the cells were transferred from the 24-well plate to 1.5 ml micro-centrifuge tubes, centrifuged at 200 rpm for 5 min and washed with 1 ml of phosphate buffered saline (PBS). The cells were lysed by incubation with passive lysis buffer (promega) for 15 min at room tempreture, harvested and cleared by centrifugation at 15,000 rpm for 3 min. Luciferase activity of three independent transfections were determined in wallac 1420 multilabel counter® (Perkin Elmer) using luciferase assay systems (promega). All transfections were performed in triplicate.

### Statistical analysis

Data were analysed with GraphPad prism (Software version 5.00, Inc; San Diego California USA). TLR9 SNPs genotype and allele frequencies were calculated by direct counting. Consistency of genotype frequency with Hardy-Weinberg Equilibrium (HWE) was determined by comparing the observed number of different genotypes with those expected under the HWE for the estimated allele frequency [24].

The incidence, attributable risk, relative risk and 95% confidence intervals (CIs) were calculated and their significances tested using t-statistics. The p value of <0.05 was considered to be significant after Bonferroni’s corrections were made for multiple testing. Briefly, the incidence of symptomatic malaria was calculated by counting the number of events in the adjusted observation period (person per year) for each genotype or haplotype. The attributable risk was calculated by subtracting the incidence of symptomatic malaria in non-carriers of a genotype/haplotype from carriers of the genotype/haplotype, while the relative risk was calculated by dividing the incidence of symptomatic malaria for a genotype/haplotype with that of non-carriers of the genotype or haplotype as previously described [25]. Statistical significance of attributable risk was evaluated by assuming null hypothesis, that attributable risk follows normal distribution N(0, SE), where SE was estimated by the formula described in reference [25].

Haplotypes for TLR9 SNPs were estimated from non-phased genotype data with maximization likelihood algorithm by the use of PHASE version 2.1.1 [26,27]. Also, pairwise linkage disequilibrium between each pair of SNP was determined using the software Haploview [28]. The heterogeneity of mean parasite density for different genotype carriers was determined by Analysis of variance (ANOVA), then the genotype effect assessed with student’s t-test comparing mean parasite density between carriers and non-carriers of each genotype.

## Results

### TLR9 genotype frequencies

**Figure 2:**
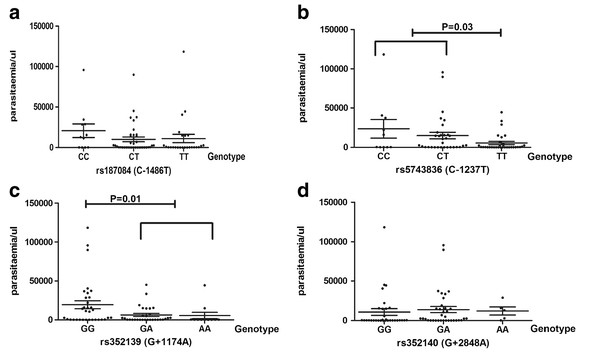
**Schematic drawing of the Toll-Like Receptor 9 gene structure and positions of the four TLR9 SNPs.** The four SNPs studied are located at position −1486, -1237, +1174 and +2848, respectively. The positions were calculated by taking the A of TLR9 ATG start codon as position 1 based on Genbank accession no. NM_17442.

In this study, four common TLR9 SNPs, (two located in the promoter region, one intronic and one in the coding region of the second exon (Figure 2)), were genotyped by direct sequencing for the 429 Ghanaian cohort children who were successfully followed up for one year. The minor allele frequencies for rs187084 (C-1486 T), rs5743836(C-1237 T), rs352139 (G + 1174A) and rs352140 (G + 2848A) were found to be 0.35, 0.38, 0.41 and 0.25, respectively, in our study population. The genotype distributions of all the SNPs examined did not show significant deviation from HWE.

### Linkage disequilibrium

Pairwise linkage disequilibrium (LD) for the four SNPs was analysed using haploview software and a weak LD was observed between the SNPs rs352139 and rs352140 (D^1^ = 1, r^2^ = 0.237); rs352139 and rs5743836 (D^1^ = 0.857, r^2^ = 0.319); and rs352139 and rs187084 (D^1^ = 0.846, r^2^ = 0.277) (Table 1).

**Table 1:** The strength of pairwise linkage disequilibrium for the four TLR9 SNPs analysed in the study

**r**^**2**^ **D**^**1**^	**rs352140**	**rs352139**	**rs5743836**	**rs187084**
**rs352140**		**0.237**	0.032	0.175
**rs352139**	**1.0**		**0.319**	**0.277**
**rs5743836**	0.4	**0.857**		0.014
**rs187084**	0.535	**0.846**	0.124	

D^1^ is the measure for deviation of the observed haplotype frequency from the expected frequency, with D^1^ =1 showing that the examined loci are completely dependent on one another. r^2^ is the correlation coefficient between pair of two loci and r^2^ > 0.4 showing a meaningful association while r^2^ > 0.2 being suggestive.

### Effect on incidence of symptomatic malaria

Assessment of the effect of TLR9 genotypes on the risk of symptomatic mild malaria was carried out by calculating the incidence of malaria, the relative risk and attributable risk for each TLR9 genotype studied (Table 2). The assessment revealed that the GG genotype for the rs352139 (G + 1174A) intronic SNP increased the attributable risk for malaria by 0.116 events per person per year, with an incidence of 0.146 vs. 0.03 for rs352139 GG vs. non-GG carriers, respectively, and a significant relative risk of 4.8 even after Bonferroni’s correction for multiple testing (p_corrected_ = 0.0024) (Table 2). In contrast, the rs352140 (G + 2848A) GG genotype decreased the attributable risk by 0.076 events per person per year, with an incidence of 0.04 vs. 0.116 for rs352140 GG vs. non-GG carriers, respectively, and a significant relative risk of 0.34 (p_corrected_ = 0.048) (Table 2). The promoter polymorphisms rs187084 (C-1486 T) and rs5743836(C-1237 T) did not show a significant influence on the incidence of symptomatic malaria in the study population (Table 2).

**Table 2:** Incidence, relative risk and attributable risk of mild malaria among carriers of TLR9 SNP’s genotypes

^**a**^**SNPs**	**Incidence**	**Relative risk**	**Attributable risk**	^**b**^**CI**	***p*value**	^**c**^**p**_**c**_
**rs187084** (C-1486 T)						
**TT** vs. CC + TC	0.04	0.37	−0.061	(−0.11)-(−0.01)	**0.01**	ns
**CC** vs. TT + TC	0.20	3.7	0.144	0.03-0.26	**0.01**	ns
**rs5743836** (C-1237 T)						
**TT** vs. CC + TC	0.04	0.38	−0.060	(−0.11)-(−0.01)	**0.01**	ns
**CC** vs. TT + TC	0.09	1.29	0.020	(−0.05)-(0.09)	0.6	ns
**rs352139** (G + 1174A)						
**GG** vs. AA + GA	**0.15**	**4.8**	**0.116**	**0.05-0.18**	**0.0003**	**0.0024**
**AA** vs. GG + GA	0.03	0.3	−0.055	(−0.10)-(−0.01)	0.03	ns
**rs352140** (G + 2848A)						
**GG** vs. AA + GA	**0.04**	**0.3**	**−0.076**	(−0.13)-(−0.02)	**0.006**	**0.048**
**AA** vs. GG + GA	0.11	1.63	0.044	(−0.08)-(0.17)	0.5	ns

^a^SNPs: Single nucleotide polymorphisms; ^b^Cl: confidence intervals; ^c^p_c_ = corrected *p* value by bonferroni method by factor of 8; ns: not statistically significant. The incidence shown is for the genotype highlighted in bold, the relative and attributable risk shown are for the same genotype when compared with the non-carriers of the genotype. Corrected p value <0.05 was considered to be statistically significant.

### Influence on parasitaemia

**Figure 3:**
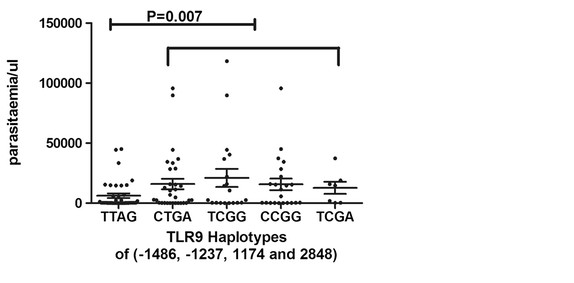
**The distribution of mean parasitaemia for carriers of the TLR9 SNP genotypes 3a): **The mean parasite load for the rs352139 (G + 1174A) GG, GA and AA genotypes are 19,376; 6,268; 5,589 parasite/μl of blood. The *GG genotype was significantly associated with higher parasitaemia compared with the non-GG genotype (19,376 vs. 6,086 parasite/μl of blood, p = 0.01). **3b):** The mean parasite load for the rs5743836 (C-1237 T) CC, CT and TT genotypes are 23,532; 14,924; 5,501 parasite/μl of blood, respectively. The *TT genotype was significantly associated with lower parasitaemia compared with the non-TT genotype (5,501 vs. 16,973 parasite/μl of blood, p = 0.03). **3c):** The mean parasite load for the rs187084 (C-1486 T) CC, CT and TT genotypes are 20,691; 10,089; 11,117 parasite/μl of blood, respectively. **3d).** The mean parasite load for the rs352140 (G + 2848A) GG, GA and AA genotypes are 10,738; 13776; 12088 parasite/μl of blood. Statistical analysis was performed using ANOVA and student’s t-test. P value of <0.05 was considered to be statistically significant.

The influence of TLR9 polymorphisms on parasitaemia was evaluated by comparing the mean parasite densities at the point of febrile episodes among carriers of each genotype. It was observed that individuals with rs352139 (G + 1174A) GG genotype had a significantly higher mean parasitaemia level compared with non-GG (GA + AA) genotype (19,376 vs. 6,086 parasite/μl, respectively, p = 0.01) (Figure 3a), while individuals with the rs5743836 (C-1237 T) TT genotype had a significantly lower mean parasite load compared with the non-TT (TC + CC) genotype (5,501 vs. 16,973 parasite/μl, respectively, p = 0.03) (Figure 3)b. No effect on mean parasitaemia was detected for the TLR9 SNPs rs187084 (C-1486 T) and rs352140 (G + 2848A) (Figure 3c and 3d).

### TLR9 haplotype analysis

Eleven (11) TLR9 haplotypes were inferred using the Phase software and their estimated haplotype frequencies in the study population are as follows; TTAG: 39.1%, CTGA: 17.3%, TCGG: 15.1%, CCGG: 14.6%, TCGG: 5.9%, TTGG: 2.6%, CCAG: 2.0%, TTGA: 1.8%, CTGG: 1.1%, TCAG: 0.2% and CTAG: 0.2% (for rs187084 (C-1486 T), rs5743836(C-1237 T), rs352139 (G + 1174A) and rs352140 (G + 2848A), respectively). The major haplotypes with frequencies greater than 5% were considered for further analysis on their possible effect on the risks for symptomatic malaria. The result revealed that the TTAG haplotype significantly reduced the attributable risk by 0.115 events per person per year, with an incidence of 0.03 vs. 0.147 for carriers of TTAG vs. non-TTAG haplotypes, respectively; and a significant relative risk of 0.22 (p_corrected_ = 4×10^-6^) (Table 3). Conversely, the CTGA haplotype significantly increased the attributable risk by 0.103 events per person per year, with an incidence of 0.147 vs. 0.044 for carriers of CTGA vs. non-CTGA haplotypes, respectively; and a significant relative risk of 3.3 (p_corrected_ = 0.005) (Table 3). The other three common haplotypes (TCGG, CCGG and TCGA) were not observed to significantly influence the risk for symptomatic malaria in our cohort study.

**Table 3:** Incidence, relative risk and attributable risk of mild malaria among carriers of TLR9 haplotypes

^**a**^**Haplotype**	**Incidence**	**Relative risk**	**Attributable risk**	^**b**^**CI**	***p*value**	^**c**^***p***_**c**_
**TTAG** vs. non-TTAG	**0.03**	**0.2**	**−0.115**	**(−0.16)-(−0.07)**	**8x10**^**-7**^	**4x10**^**-6**^
**CTGA** vs. non-CTGA	**0.15**	**3.3**	**0.103**	**0.04-0.16**	**0.001**	**0.005**
**TCGG** vs. non TCGG	0.10	1.5	0.032	(−0.03)-(0.09)	0.3	ns
**CCGG** vs. non-CCGG	0.12	2.1	0.063	0.0-0.1	0.05	ns
**TCGA** vs. non-TCGA	0.09	1.2	0.012	(−0.07)-(0.09)	0.8	ns

^a^Haplotype for rs187084 (C-1486 T), rs5743836 (C-1237 T), rs352139 (G + 1174A) and rs352140 (G + 2848A), respectively; ^b^Cl: confidence intervals; ^**c**^*p*_c_ = corrected *p* value by Bonferroni’s method by a factor of 5; ns: not statistically significant. The incidence shown is for the haplotype highlighted in bold, the relative and attributable risk shown are for the same haplotype when compared with the non-carriers of the haplotype. Corrected p value <0.05 was considered to be statistically significant.

**Figure 4:**
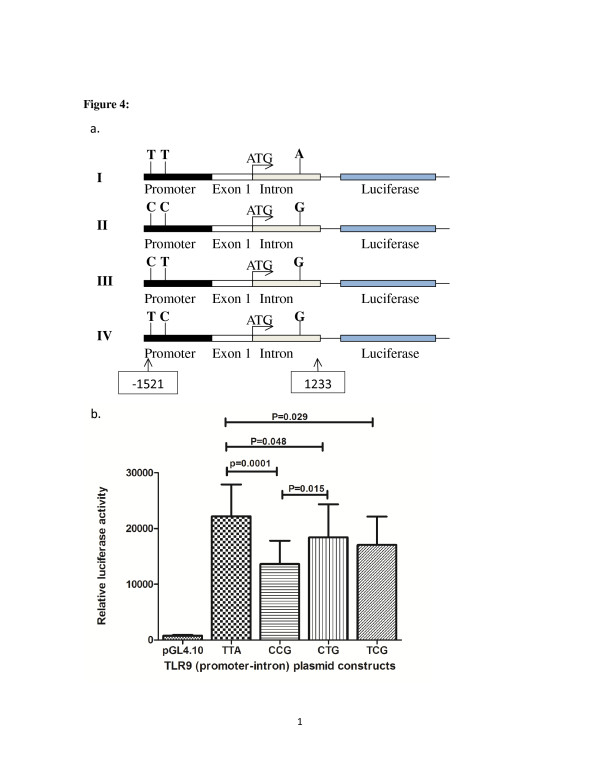
**The distribution of mean parasitaemia for carriers of the TLR9 haplotypes.** The mean parasite load for the haplotypes are TTAG, CTGA, TCGG, CCGG and TCGA are 6,178; 15,992; 21,034; 15,631; 12,714 parasite/μl of blood. The *TTAG haplotype was significantly associated with lower parasitaemia compared with the non-TTAG haplotype (6,178 vs. 20,114 parasite/μl of blood, p = 0.007). Statistical analysis was performed using ANOVA and student’s t-test. P value of <0.05 was considered to be statistically significant.

On analysing the effect of TLR9 haplotypes on parasitaemia, it was observed that the TTAG haplotype was significantly associated with lower mean parasitaemia, with carriers of TTAG having a mean parasite load of 6,178 parasite/μl compared with 20,114 parasite/μl of the non-TTAG carriers (p = 0.007) (Figure 4). No significant effect on parasitaemia was observed for the haplotypes CTGA, TCGG, CCGG and TCGA in our study population (Figure 4).

### Luciferase reporter gene assay

To further examine the functional relevance of the haplotypes in terms of the TLR9 gene transcription, four promoter plasmids containing the TLR9 promoter, exon 1 and intron 1 regions were prepared (Figure 1a) and transfected into THP-1 cells. The four constructs, represents the five major TLR9 haplotype in our population, with TTA, CTG, TCG, and CCG SNPs at positions −1486, -1237 and +1174, respectively (Figure 1a). All the four constructs consistently showed a significant higher relative luciferase activity than the negative control (empty pGL4.10 vector). It was observed that the TTA promoter plasmid has a significantly higher luciferase activity compared with all the other three promoter plasmids, i.e. the CCG, CTG and TCG (P = 0.0001, P = 0.048 and P = 0.029, respectively) (Figure 1b). It was also observed that the CTG promoter plasmid had a significantly higher luciferase activity than the CCG promoter plasmid (P = 0.015) (Figure 1b).

## Discussion

The TLR9 polymorphisms have been postulated to have a cis-regulatory effect on TLR9 expression [20] and also shown to alter cytokine levels during severe malaria infections [29]. In this study, the effect of TLR9 gene polymorphisms on symptomatic malaria was investigated and TLR9 polymorphisms and haplotypes were significantly associated with susceptibility to symptomatic malaria among Ghanaian children.

In this cohort study, the intronic polymorphism rs352139 (G + 1174A) GG genotype was significantly associated with increased risk for symptomatic malaria and high parasitaemia. Previous functional study has reported that this intronic SNP has a regulatory effect on TLR9 expression, with the rs352139 G allele in combination with the promoter rs187084 (C-1486 T) C allele having a down-regulatory effect, while the rs352139 A allele in combination with rs187084 T allele having an up-regulatory effect on TLR9 expression among patients with systemic lupus erythematosus [30]. However, it is currently not clear how this intronic SNP induces such a phenotype change. It is possible that it influences signalling by creating an alternative splicing site and thus, affecting the mRNA transcription and the final protein product. Equally, the rs352139 SNP could be a likely marker in LD with a polymorphic regulatory region that controls TLR9 expression or a functional coding region SNP. In contrast to our finding, Campino *et al*[20] reported an association of the rs352139 A allele with severe malaria among Malawian population but not in the Gambian population [20]. This discrepancy may in part be explained by the difference in phenotypes and populations examined, and also by the different role pro-inflammatory cytokines play in the pathogenesis of symptomatic and severe malaria. For example, an early high IFN-γ response has been reported to confer protection against symptomatic malaria episodes [31,32], while an over-production of IFN-γ has been associated with susceptibility to cerebral malaria [33].

The synonymous coding SNP, rs352140 (G + 2848A) GG genotype was associated with protection from symptomatic malaria but no such protective effect was observed for parasitaemia among our cohort population. This rs352140 SNP is linked to the rs352139 (G + 1174A) (D^1^ = 1, r^2^ =0.237) in the study population, with the rs352140 G allele linked to rs352139 A allele. Thus, the observed protective effect could partly be attributed to the effect of the rs352139 A allele which has been shown to have an up-regulatory effect on TLR9 expression [30].

The promoter polymorphism rs5743836 (C-1237 T) TT genotype was associated with low parasitaemia but no effect on susceptibility to symptomatic malaria was observed in this study. Our finding is consistent with that of Leorrati *et al*[19] who reported an association of rs5743836 TT genotype with low parasitaemia (<10000 parasite/μl) among adults with mild malaria in the Brazilian Amazon [19]. The rs5743836 TT variant has been shown to have a higher promoter activity than the CC genotype [34], and thus, could result in increased pro-inflammatory cytokine production during malaria infection leading to successful control and elimination of malaria parasites.

On haplotype analysis, the TLR9 TTAG (−1486 T, -1237 T, +1174A, +2848 G) haplotype, the most frequent haplotype (39.1%) in the study population, was significantly associated with protection against symptomatic malaria and high parasitaemia. These findings were strongly supported by the result of luciferase reporter gene expression assay which showed a significantly higher promoter activity for the TTA (−1486 T, -1237 T, +1174A) haplotype compared with the CCG, CTG and TCG haplotypes (Figure 1b). It was also observed that the CTG haplotype had a higher promoter activity than the CCG haplotype (Figure 1b). Taken together, the haplotype with A allele for rs352139 (TTA) consistently had a significantly higher promoter effect than all the haplotypes with G allele for the same SNP (i.e. CC**G**, CT**G** and TC**G**). It was also observed that the haplotypes with T allele for rs5743836 (i.e. T**T**A, C**T**G) had a higher promoter effect than those with C allele for the same SNP (i.e. C**C**G, T**C**G) and that carriers of the T**TA** haplotype had the highest promoter activity, the C**TG** haplotype had an intermediate activity, and the C**CG** having the lowest promoter activity. Thus, it is possible that these two SNPs (the rs5743836 (C-1237 T) and rs352139 (G + 1174A)) have a greater role of influencing TLR9 gene transcription and expression. This luciferase assay result is in agreement with the findings of Tao *et al* 2007, who reported that the rs187084 (C-1486 T) T allele in combination with rs352139 (G + 1174A) A allele had a higher TLR9 expression compared with the rs187084 C allele in combination with rs352139 G allele among Japanese population who are not polymorphic for rs5743836 (C-1237 T) (i.e. TTA had higher TLR9 expression than CTG) (31). Therefore, it can be inferred that the TTAG haplotype may exert an increased TLR9 transcriptional activity resulting in higher TLR9 expression and subsequently higher pro-inflammatory cytokine production, thus conferring protection against symptomatic malaria phenotype. The TTAG haplotype has also been associated with increased risk for meningococcal meningitis among Dutch children [35] and also associated with protection from ulcerative colitis among the Japanese population [36]. The different effects of TLR9 haplotypes in different disease phenotypes could be explained by the different roles inflammatory cytokines play in pathogenesis of these diseases. While robust pro-inflammatory cytokines has been reported to provide protection against clinical mild malaria [31] and against experimental colitis in mice [37]; in bacterial meningitis robust inflammatory response has been identified as harmful and contributing to tissue damage [38].

The major limitation of this study is the low incidence of symptomatic malaria [22]. The study was conducted in an area with low but perennial malaria transmission with the intention of identifying clearly individuals susceptible or resistant to the symptomatic malaria phenotypes. Nevertheless, this study has highlighted clearly that the TLR9 gene polymorphisms significantly influence susceptibility to symptomatic malaria. It is therefore worthwhile to carry out more studies in areas with different malaria transmission and using a larger sample size, so that the role of TLR9 polymorphisms on different malaria phenotypes can clearly be deciphered.

## Abbreviations

TLR, Toll like receptor; SNPs, Single nucleotide polymorphisms; WHO, World Health Organization; HWE, Hardy-Weinberg’s Equilibrium; LD, Linkage disequilibrium.

## Competing interests

The authors declare that they have no competing interests.

## Authors’ contributions

AHO participated in conception and designing of the study, carried out the molecular genetic studies, the luciferase reporter gene assay, performed data analysis, interpretation, and drafted the manuscript; YM participated in conception and designing of the study, data analysis, interpretation and revising the manuscript critically; YA participated in designing of the study, data collection and analysis; HS assisted during the luciferase reporter gene assay experiment; MFO and BDA participated in designing of the study, and data collection; SMN participated in designing of the study and revising manuscript critically; KM participated in designing of the study and data analysis; KH participated in conception and designing of the study, data analysis, interpretation, revising manuscript critically and gave final approval of the version to be published. All authors read and approved the final manuscript.
